# Sliding Mode Tracking Control of a Wire-Driven Upper-Limb Rehabilitation Robot with Nonlinear Disturbance Observer

**DOI:** 10.3389/fneur.2017.00646

**Published:** 2017-12-04

**Authors:** Jie Niu, Qianqian Yang, Xiaoyun Wang, Rong Song

**Affiliations:** ^1^Guangdong Provincial Engineering and Technology Center of Advanced and Portable Medical Devices, School of Engineering, Sun Yat-sen University, Guangzhou, China; ^2^Injury Rehabilitation Hospital of Guangdong Province, Guangzhou, China

**Keywords:** rehabilitation robot, wire-driven, upper limb, tracking control, sliding mode, nonlinear disturbance observer

## Abstract

Robot-aided rehabilitation has become an important technology to restore and reinforce motor functions of patients with extremity impairment, whereas it can be extremely challenging to achieve satisfactory tracking performance due to uncertainties and disturbances during rehabilitation training. In this paper, a wire-driven rehabilitation robot that can work over a three-dimensional space is designed for upper-limb rehabilitation, and sliding mode control with nonlinear disturbance observer is designed for the robot to deal with the problem of unpredictable disturbances during robot-assisted training. Then, simulation and experiments of trajectory tracking are carried out to evaluate the performance of the system, the position errors, and the output forces of the designed control scheme are compared with those of the traditional sliding mode control (SMC) scheme. The results show that the designed control scheme can effectively reduce the tracking errors and chattering of the output forces as compared with the traditional SMC scheme, which indicates that the nonlinear disturbance observer can reduce the effect of unpredictable disturbances. The designed control scheme for the wire-driven rehabilitation robot has potential to assist patients with stroke in performing repetitive rehabilitation training.

## Introduction

Stroke, an acute cerebrovascular disease typically caused by hemorrhage or blockage in brain blood vessels, is a major cause of motor dysfunction or even permanent disability (1). Repetitive training and task-oriented exercises of the paretic extremity are significantly beneficial to the recovery of extremity functions (2–4). However, traditional therapy for the rehabilitation relies heavily on the experience and manual manipulation of physical therapists, which is time-consuming, labor-intensive, and costly. Robot-aided rehabilitation has many advantages such as high efficiency, high precision, and controllability (5–7), which can allow more intensive training (8, 9) and reduce the workload of physical therapists.

Mechanical structure is one of the most important factors that can affect the effectiveness of the robot-assisted rehabilitation. In the past two decades, many robotic systems have been designed and applied in rehabilitation training. Conventional rehabilitation robots usually consist of several rigid links. MIT-MANUS has two degrees-of-freedom (DOF) and can guide the upper-limb over a horizontal plane (5). Mirror-image movement enabler is an upper-limb rehabilitation robot that can ensure the hemiparetic arm move to the mirror-image position of the opposite arm in a three-dimensional space (8). Fazekas et al. have designed a robotic system based on two industrial robots for upper-limb rehabilitation to assist three-dimensional movements (10). Though these rehabilitation robots can be used for rehabilitation training, they have many limitations like poor workspace, lack of compliance, and relatively high construction costs. Wire-driven rehabilitation robots not only remedy these shortcomings but have simple structures and are easy to assemble/disassemble. Moreover, since the wire-driven rehabilitation robots have lower moving inertia compared to rehabilitation robots that consist of rigid links, the user can feel less constraint and more comfort when participating in robot-assisted rehabilitation training. In the past few years, some wire-driven robots have been designed for rehabilitation. Jones et al. have designed a wire-actuated rehabilitation robot, which is a three DOF robotic exoskeleton for hand rehabilitation (11). Sophia-3, an end-effector based wire-driven rehabilitation robot, has been designed to assist planar movements (12). Gaponov et al. have designed a portable cable-driven rehabilitation robot, which provides assistance for shoulder, abduction, and elbow flexion (13).

Control scheme is another important factor affects the effectiveness of robot-assisted rehabilitation. When delivering task-oriented rehabilitation training, the control scheme is required to assist the robot in guiding the paretic limb to finish predefined movements or trajectory accurately and compliantly. Linear control techniques such as PID (14, 15) and PD (15, 16) controllers have been designed for rehabilitation robot, but they have degraded performance if nonlinear uncertainties of the system are considered (17). Simple nonlinear control techniques such as robust torque control scheme (14, 15) and impedance control scheme (15, 18) cannot meet the requirement under uncertain dynamics. Many other control schemes have been presented such as fuzzy adaption (19) and adaptive control schemes (17, 18), whereas these control schemes perform well for industrial robots but not for rehabilitation robots due to uncertainties and disturbances in rehabilitation training (20). Sliding mode control (SMC) is a variable structure control method, which has inherent insensitivity and robustness against uncertainties and disturbances. Therefore, it can be relatively suitable for the control of human–robot interaction systems. However, to achieve satisfactory tracking performance, traditional SMC needs high-control authority to eliminate model uncertainties and external disturbances, which in turn is the main cause of chattering (21). Chattering is undesirable since it may degrade tracking performance and even cause damage to actuators (22). A relatively easy and effective approach to address this problem is to employ a nonlinear disturbance observer in the control loop to estimate all the lumped uncertainties and disturbances. By this means, the tracking precision can be improved since uncertainties and disturbances can be estimated and compensated for, while the chattering phenomenon can be reduced since the control scheme may not need high-control authority to resist disturbances. Though nonlinear disturbance observer has been investigated in various fields (23, 24), to the largest of our knowledge, it is rarely utilized in a wire-driven rehabilitation robot.

In this paper, a wire-driven rehabilitation robot is designed to deliver task-oriented training exercises for upper-limb, which can work over a three-dimensional space. First, sliding mode control with nonlinear disturbance observer (SMCNDO) is designed for the wire-driven rehabilitation robot against unpredictable disturbances. Then, simulation of tracking a predefined trajectory is conducted to investigate the performance of the designed control scheme. Moreover, a square-shaped and a circle-shaped trajectory are designed, and the forearm of the subject is controlled by the robot to follow the predefined trajectories. Both simulation and experimental results of the designed SMCNDO are compared with that of a traditional SMC.

## Materials and Methods

### System Description

**Figure 1:**
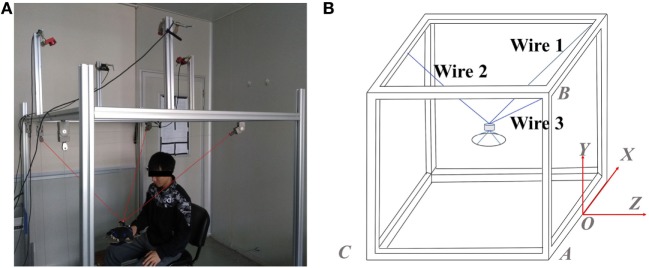
A wire-driven rehabilitation robot. **(A)** Mechanical architecture of the wire-driven rehabilitation robot and **(B)** geometry of the wire-driven rehabilitation robot.

Mechanical architecture of the designed wire-driven rehabilitation robot is shown in Figure 1A, which consists of three actuated wire-driven limbs to perform translational movement in a three-dimensional space. The main software program provides control scheme, reference trajectory, and data storage procedures, which is written in VC++. In addition, a personal computer is used to perform real-time processing and real-time communication with I/Os and serves as a user interface that can be used to manage the system. Motion capture system is implemented to ensure relatively accurate measurement. From Figure 1A, four cameras are attached on the top of the base frame, and a spherical marker is placed on the top of the end-effector. The position of the marker can be directly obtained by motion capture system, which is used to represent the position of the end-effector and as the feedback in the control loop. Safety is an important factor that needs to be considered. To ensure security and prevent accidents during rehabilitation training, emergency stop switches are designed both in software and hardware. When a patient feels uncomfortable or any accident happens, the power supply for the system can be immediately interrupted by pressing software or hardware emergency stop switch. Moreover, the output force of each actuator is strictly limited when designing software program.

### Control Scheme Design

The geometry of the designed wire-driven rehabilitation robot is shown in Figure 1B. The origin of the coordinate system is chosen referring to the midpoint of a certain side of the cube-shaped structure, which is shown in Figure 1B. The position of the top of each winch pulley can be obtained by measurement. The end-effector is simply treated as a point mass, and its position can be obtained directly by the implementation of the task space coordinates, and it is assumed that the end-effector is lying inside the workspace. Therefore, the length of each wire can be calculated according to the spatial position of the end-effector and the spatial position of related winch pulley:

(1) $$L_{i}=\sqrt{{\left(x-x_{i}\right)}^{2}+{\left(y-y_{i}\right)}^{2}+{\left(z-z_{i}\right)}^{2}}$$

where (*x, y, z*) represents the position of the end-effector, *i* varying from 1 to the number of wires, and (*x_i_, y_i_, z_i_*) represents the position of the associated *i*th winch pulley.

According to Table 1, (*x_i_, y_i_, z_i_*) can be expressed specifically as follows:

(2) $$\left\{\begin{array}{l} \left(x_{1},y_{1},z_{1}\right)=\left(a,b,0\right)\;\\ \left(x_{2},y_{2},z_{2}\right)=\left(0,b,-c\right)\;\\ \left(x_{3},y_{3},z_{3}\right)=\left(-a,b,0\right)\; \end{array}\right.$$

where *a* represents the length of |*OA*|, *b* represents the length of |*AB*|, and *c* represents the length of |*AC*|. Therefore, from Eqs 1 and 2, the length of the wires can be written exactly as follows:

(3) $$\left\{\begin{array}{c} L_{1}=\sqrt{{\left(x-a\right)}^{2}+{\left(y-b\right)}^{2}+z^{2}}\\ L_{2}=\sqrt{x^{2}+{\left(y-b\right)}^{2}+{\left(z+c\right)}^{2}}\\ L_{3}=\sqrt{{\left(x+a\right)}^{2}+{\left(y-b\right)}^{2}+z^{2}} \end{array}\right..$$

**Table 1:** Parameters of the designed wire-driven rehabilitation robot.

Parameter	Symbol	Value
End-effector mass	*m*	2.5 kg
The length of *|OA|*	*a*	0.81 m
The length of *|AB|*	*b*	1.78 m
The length of *|AC|*	*c*	1.36 m
Gravity acceleration	*g*	9.8 m/s^2^

Differentiating the length of each wire with respect to time leads to velocity of each wire:

(4) $$\left\{\begin{array}{l} {\dot{L}}_{1}=\left[\dot{x}\left(x-a\right)+\dot{y}\left(y-b\right)+\dot{z}z\right]∕L_{1}\;\\ {\dot{L}}_{2}=\left[\dot{x}x+\dot{y}\left(y-b\right)+\dot{z}\left(z+c\right)\right]∕L_{2}\;\\ {\dot{L}}_{3}=\left[\dot{x}\left(x+a\right)+\dot{y}\left(y-b\right)+\dot{z}z\right]∕L_{3}\; \end{array}\right..$$

Moreover, the relation between ${\dot{L}}_{i}$ and $\dot{x}$, $\dot{y}$, and $\dot{z}$ can be established based on Jacobian matrix. Jacobian matrix for the designed wire-driven rehabilitation robot can be expressed specifically as follows:

(5) $$\begin{array}{ll} \text{J} & =\left[\begin{array}{ccc} \partial L_{1}∕\partial x & \partial L_{1}∕\partial y & \partial L_{1}∕\partial \text{z}\\ \partial L_{2}∕\partial x & \partial L_{2}∕\partial y & \partial L_{2}∕\partial \text{z}\\ \partial L_{3}∕\partial x & \partial L_{3}∕\partial y & \partial L_{3}∕\partial z \end{array}\right]\\ & =\left[\begin{array}{ccc} \left(x-a\right)∕L_{1} & \left(y-b\right)∕L_{1} & z∕L_{1}\\ x∕L_{2} & \left(y-b\right)∕L_{2} & \left(z+c\right)∕L_{2}\\ \left(x+a\right)∕L_{3} & \left(y-b\right)∕L_{3} & z∕L_{3} \end{array}\right]. \end{array}$$

Moreover, in this study, we assume that the motion is within the workspace and the gravity of the end-effector is able to keep all the wires under tension. Therefore, the relation between externally applied forces and the wire tensile forces can be expressed as follows (25):

(6) $${\left[\begin{array}{ccc} u_{x} & u_{y} & u_{z} \end{array}\right]}^{\text{T}}=-{\text{J}}^{\text{T}}\tau$$

where τ denotes the vector of wire-driven forces. In this study, the dynamic equation of the wire-driven rehabilitation robot can be expressed as follows (25):

(7) $$\text{M}\left(\ddot{\text{p}}\right)\ddot{\text{p}}+\text{C}\left(\text{p},\dot{\text{p}}\right)\dot{\text{p}}+\text{G}\left(\text{p}\right)=\text{u}+\text{d}$$

where $\text{M}\left(\ddot{\text{p}}\right)$ denotes the inertia matrix, $\text{C}\left(\text{p},\dot{\text{p}}\right)$ is the vector of Coriolis and centripetal terms, ***G****(****p****)* is the vector of gravity terms, and ***d*** denotes the vector of all the lumped uncertainties (parametric, model, and disturbance). In this study, the designed wire-driven rehabilitation robot is considered to perform only translational movements, low velocities, and accelerations are involved in rehabilitation training, therefore Coriolis forces can be neglected. Hence, Eq. 7 can be simplified as follows:

(8) $$\text{M}\left(\text{p}\right)\ddot{\text{p}}+\text{G}\left(\text{p}\right)=\text{u}+\text{d}$$

where

$$\text{M}=\left[\begin{array}{ccc} m & 0 & 0\\ 0 & m & 0\\ 0 & 0 & m \end{array}\right],\;\text{G}=\left[\begin{array}{c} 0\\ \mathrm{mg}\\ 0 \end{array}\right],\;\text{d}=\left[\begin{array}{c} d_{x}\\ d_{y}\\ d_{z} \end{array}\right]$$

*d_x_, d_y_*, and *d_z_* represent the disturbances in *X, Y*, and *Z* directions. Therefore, referring to Eqs 6 and 8, wire cable-driven forces in terms of ***p*** generalized coordinates can be obtained as:

(9) $$\tau =-{\left({\text{J}}^{\text{T}}\right)}^{-1}\left(\text{M}\left(\text{p}\right)\ddot{\text{p}}+\text{G}\left(\text{p}\right)-\text{d}\right).$$

Note that ***p* ** = [*x y z*]^T^ and ***p_d_* ** = [*x_d_ y_d_ z_d_*]^T^ represents the actual and the desired position in a three-dimensional space, respectively. For the sake of control scheme design, the vector of sliding surfaces is defined as follows:

(10) $$\text{s}=\lambda \text{e}+\dot{\text{e}}$$

where ***e* ** = [*x*−*x_d_ x*−*x_d_ x*−*x_d_*]^T^ is the vector of tracking errors in a three-dimensional space, ***s* ** = [*s_x_ s_y_ s_z_*]^T^ and ***λ*** = [λ*_x_* λ*_y_* λ*_z_*]^T^ are positive symmetric diagonal matrices. Taking the derivative of ***s*** leads to $\dot{\text{s}}$. And $\dot{\text{s}}$ is the so-called reaching law, and in this study it can be defined as follows:

(11) $$\dot{\text{s}}=-\text{ks}-\eta \text{sgn}\left(\text{s}\right)-{\text{M}}^{-1}\;\tilde{\text{d}}$$

where sgn(***s***) is a discontinuous function, it can be given as follows:

(12) $$\text{sgn}\left(s_{i}\right)=\left\{\begin{array}{ll} \frac{s_{i}}{∥s_{i}∥},\text{when}\; & ∥s_{i}∥>0\\ 0,\text{ when}\; & ∥s_{i}∥\text{=}0 \end{array}\right.$$

and sgn(***s***) = [sgn(*s_x_*) sgn(*s_y_*) sgn(*s_z_*)]^T^, $\tilde{\text{d}}$ denotes the vector of the estimation errors which is given as $\tilde{\text{d}}=\text{d}-\;\hat{\text{d}}$, ***k* ** = diag(*k_x_ k_y_ k_z_*) and **η** = diag(η*_x_* η*_y_* η*_z_*) are symmetric diagonal positive matrices need to be designed in practice. In this study, a switching sliding surface is considered by defining control gain as follows:

(13) $$\eta_{i}=\left\{\begin{array}{l} \eta_{1},\begin{array}{cc} \text{when} & ∥s_{i}∥\leq \beta \end{array}\;\\ \eta_{2},\begin{array}{cc} \text{when} & ∥s_{i}∥>\beta \end{array}\; \end{array}\right.\;,\;i=x,y,z.$$

According to Eqs 9–11, through a series of substitutions and transformations, the SMC scheme can be expressed as follows:

(14) $$\text{u}=\text{M}\left(\lambda \dot{\text{e}}+{\ddot{\text{p}}}_{d}+\text{ks}+\eta \text{sgn}\left(\text{s}\right)\right)+\text{G}-\;\hat{\text{d}}.$$

A nonlinear disturbance observer has been introduced in many studies (26–28). In this study, a nonlinear disturbance observer is designed for the wire-driven robot to estimate and compensate disturbances and uncertainties that exist in the rehabilitation training, which can be defined as follows:

(15) $$\hat{\text{d}}=\text{q}+\gamma \text{M}\dot{\text{p}}$$

(16) $$\dot{q}=\gamma \left(\text{G}-\text{u}-\;\hat{\text{d}}\right)$$

where ***d*** is estimated as $\hat{\text{d}}$, ***q*** is an auxiliary vector, ***γ*** = diag(γ*_x_* γ*_y_* γ*_z_*) is a symmetric diagonal positive matrix. In this study, we consider that the disturbances are slow time-varying signal, which means $\dot{\text{d}}=\text{0}$. According to Eqs 14–16, through a series of translations, we can find that $\dot{\tilde{\text{d}}}+\gamma \tilde{\text{d}}=\text{0}$, therefore, it can be noticed that the estimation errors will convergence to 0 exponentially by using the designed nonlinear disturbance observer. Furthermore, to prove the closed-loop stability of the control scheme, the Lyapunov function is chosen as follows:

(17) $$V=\frac{1}{2}{\text{s}}^{\text{T}}\text{s}+\frac{1}{2}{\;\tilde{\text{d}}}^{\text{T}}\;\tilde{\text{d}}.$$

The derivative of *V* is:

(18) $$\dot{V}={\text{s}}^{\text{T}}\dot{\text{s}}+{\;\tilde{\text{d}}}^{\text{T}}\;\;\dot{\tilde{\text{d}}}.$$

Substitute Eq. 11 and $\dot{\tilde{\text{d}}}+\gamma \tilde{\text{d}}=\text{0}$ into the Eq. 18, one can notice that:

(19) $$\dot{V}=-{\text{s}}^{\text{T}}\text{k}\dot{\text{s}}-\eta ∥\text{s}∥-{\text{s}}^{\text{T}}{\text{M}}^{-1}\;\tilde{\text{d}}-{\;\tilde{\text{d}}}^{\text{T}}\gamma \;\tilde{\text{d}}.$$

Therefore, $\dot{V}$ can be rewritten as:

(20) $$\dot{V}=-{\text{s}}^{\text{T}}\text{ks}-{\;\tilde{\text{d}}}^{\text{T}}\gamma \;\tilde{\text{d}}-\sum \left(\eta_{i}|s_{i}|+s_{i}{\;\tilde{d}}_{i}∕m\right),i=x,y,z.$$

In this study, $|{\tilde{d}}_{i}{|}_{\max}$ represents the maximum value of $\left|{\;\tilde{d}}_{i}\right|$, and we assume η*_i_* satisfies $\eta_{i}\geq |{\tilde{d}}_{i}{|}_{\max}∕m,i=x\text{,}y\text{,}z$. Therefore, $\dot{V}$ can be expressed as follows:

(21) $$\dot{V}\leq -{\text{s}}^{\text{T}}\text{ks}-{\;\tilde{\text{d}}}^{\text{T}}\gamma \;\tilde{\text{d}}.$$

As mentioned previously that ***k*** and ***γ*** are symmetric diagonal positive matrices, therefore, one can notice that $\dot{V}\leq 0$. Therefore, the Lyapunov function is always decreasing, which means the closed-loop system is asymptotically stable. As described previously that sgn(***s***) function is a discontinuous function, the control law is discontinuous across the sliding surface, which may cause chattering. Chattering is undesirable since it can cause damage to the actuators being controlled. Chattering reduction can be achieved by using a nonlinear disturbance observer to estimate and compensate disturbances; furthermore, chattering can be reduced by replacing the discontinuous sgn(***s***) function with a continuous sat(***s***) function. Using sat(***s***) function, the discontinuity of the controller is smoothed in a thin boundary layer neighboring the sliding surface. sat(***s***) function is defined as follows:

(22) $$\mathrm{sat}\left(s_{i}\right)=\left\{\begin{array}{l} \frac{s_{i}}{∥s_{i}∥},\begin{array}{cc} \text{when} & ∥s_{i}∥>\Delta \end{array}\;\\ \frac{s_{i}}{\Delta },\begin{array}{cc} \text{when} & \;∥s_{i}∥<\Delta \end{array}\; \end{array}\right.\;,\;i=x,y,z$$

where Δ is a small and positive constant. Therefore, control scheme for the wire-driven forces by the SMC scheme with nonlinear disturbance observer can be rewritten as:

(23) $$\tau =-{\left({\text{J}}^{\text{T}}\right)}^{-1}\left(\text{M}\left(\lambda \dot{\text{e}}+{\ddot{\text{p}}}_{d}+\text{ks}+\eta \text{sat}\left(\text{s}\right)\right)+\text{G}-\;\hat{\text{d}}\right).$$

### Simulation and Experimental Setup

To investigate the effectiveness of the presented SMCNDO for the wire-driven rehabilitation robot, first, simulation is carried out in Matlab/Simulink. Both SMCNDO and the traditional SMC are considered in the simulation, and the results of tracking a predefined trajectory *via* SMCNDO are compared with that of the traditional SMC. Parameters for the wire-driven rehabilitation robot are shown in Table 1. The reference trajectory is predefined as ${\text{p}}_{d}\left(t\right)={\left[\begin{array}{ccc} 0.1\sin\left(0.1t\right) & 0.98 & 0.1\cos\left(0.1t\right)-0.52 \end{array}\right]}^{\text{T}}$. In addition, the vector of the desired velocities and accelerations along the three coordinate directions can be obtained by differentiating ***p****_d_*(*t*) once and twice, respectively. The vector of disturbances along three coordinate directions is defined as follows:

(24) $$\text{d}\left(t\right)={\text{d}}_{1}(t)+{\text{d}}_{2}\left(t\right)$$

where ***d****_1_*(*t*) = [5sin(*t*) 5sin(*t*) 5sin(*t*)]_T_, ***d****_2_*(*t*) is a vector of pulse-like disturbances which is defined as follows:

(25) $${\text{d}}_{2}\left(t\right)=\left\{\begin{array}{c} {\left[\begin{array}{ccc} -200 & -200 & 200 \end{array}\right]}^{\text{T}},\\ {\left[\begin{array}{ccc} 200 & 200 & -200 \end{array}\right]}^{\text{T}},\\ {\left[\begin{array}{ccc} -200 & -200 & 200 \end{array}\right]}^{\text{T}}, \end{array}\right.\begin{array}{c} \begin{array}{cc} \;\text{if} & 0\leq t<15\text{s} \end{array}\\ \begin{array}{cc} \;\text{if} & 15\text{s}\leq t<45\text{s} \end{array}\\ \begin{array}{cc} \;\text{if} & 45\text{s}\leq t<62.8\text{s} \end{array} \end{array}.$$

Both the traditional SMC and SMCNDO can be designed according to Eq. 23, and $\hat{\text{d}}$ term is considered as 0 for the traditional SMC. A nonlinear disturbance is designed based on Eqs 15 and 16. According to the design procedures, proper control parameters for SMC are given as: ***λ*** = diag(10,10,10), ***k*** *=* diag(5,5,5), η_1_ = 0.1, η_2_ = 80, β = 0.5, and proper control parameters for SMCNDO are given as: ***λ*** = diag(10,10,10), ***k* ** = diag(5,5,5), ***γ*** = diag(50,50,50), η_1_ = 0.1, η_2_ = 80, β = 0.5.

**Figure 2:**
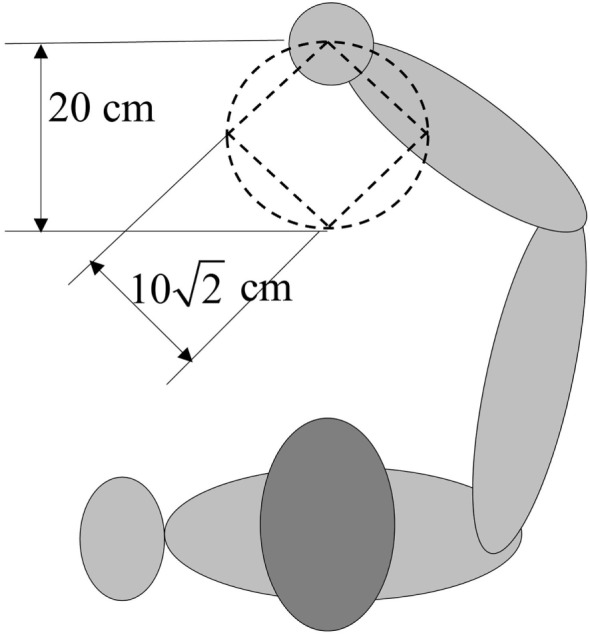
Predefined square-shaped and circle-shaped trajectories in experiments. The side length of square-shaped trajectory is $\text{0.1}\sqrt{\text{2}}\text{ m}$, and the radius of the circle is 0.1 m.

Experiments are carried out based on a real-time system to verify the performance of the presented controller for practical applications. It has been approved by the ethics committee of the Injury Rehabilitation Hospital of Guangdong Province. During the experiments, a healthy subject (23 years old, height of 173 cm, and weight of 60 kg) is seated beside the wire-driven rehabilitation robot with his forearm placed on the splint. The subject is instructed to keep relaxed during the experiments. Then, the forearm of the subject is controlled by the robot to follow predefined trajectories, and two different types of trajectories are considered in this section, including a square-shaped and a circle-shaped trajectory. The results of SMCNDO are compared with that of the traditional SMC. The traditional SMC and SMCNDO are designed according to Eq. 23 and $\hat{\text{d}}$ is also considered as 0 for the traditional SMC, which is the same as previous simulation. A nonlinear disturbance observer is also designed based on Eqs 15 and 16. According to the design procedures and considering the practical application, proper control parameters for SMC and SMCNDO are given as: ***λ*** = diag(55,55,55), ***k* ** = diag(20,20,20), η_1_ = 2,000, η_2_ = 500, β = 0.1, and proper control parameters for SMCNDO is chosen as: ***λ*** = diag(55,55,55), ***k* ** = diag(20,20,20), η_1_ = 2,000, η_2_ = 500, β = 0.1, ***γ*** = diag(3,3,3). The square-shaped trajectory which is predefined as the reference trajectory for the experiment is shown in Figure 2, and the side length is $0.1\sqrt{2}$ meters. Each experiment of tracking the square-shaped trajectory lasts 60 s. And the circle-shaped reference trajectory with a radius of 0.1 m is predefined as ${\text{p}}_{d}\left(t\right)={\left[\begin{array}{ccc} 0.1\sin\left(0.1t\right) & 0.98 & 0.1\cos\left(0.1t\right)-0.52 \end{array}\right]}^{\text{T}}$.

The desired velocity and acceleration can be obtained by differentiating the desired position once and twice, respectively, each experiment of tracking the circle-shaped trajectory lasts 62.8 s. The predefined circle-shaped trajectory is visually shown in Figure 2. The root mean square errors are calculated to evaluate the tracking accuracy of the two applied control schemes when tracking the two predefined trajectories. The root mean square error between actual position and desired position is used to evaluate the tracking accuracy, which can be expressed as follows:

(26) $$\text{RMSE}=\sqrt{\frac{1}{N}\sum_{i=1}^{N}{\left(\delta_{d}\left(i\right)-\delta \left(i\right)\right)}^{2}},\;\delta =x,y,z.$$

## Results

**Figure 3:**
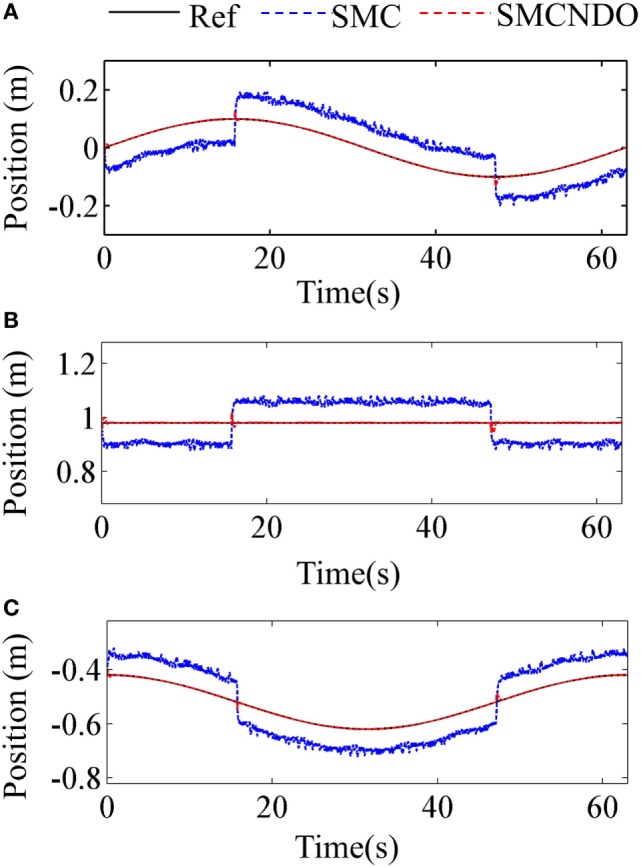
Tracking performance in the three coordinate directions *via* sliding mode control (SMC) and sliding mode control with nonlinear disturbance observer (SMCNDO). **(A)** Tracking performance in *X* direction, **(B)** tracking performance in *Y* direction, and **(C)** tracking performance in *Z* direction.

**Figure 4:**
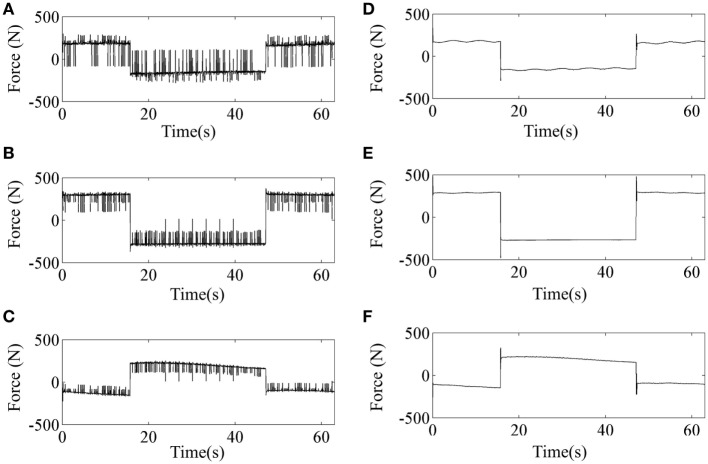
The output forces for the three wires *via* sliding mode control (SMC) and sliding mode control with nonlinear disturbance observer (SMCNDO). **(A)** The output force for wire 1 *via* SMC, **(B)** the output force for wire 2 *via* SMC, **(C)** the output force for wire 3 *via* SMC, **(D)** the output force for wire 1 *via* SMCNDO, **(E)** the output force for wire 2 *via* SMCNDO, and **(F)** the output force for wire 3 *via* SMCNDO.

Tracking performance of the traditional SMC and SMCNDO in simulation are shown in Figure 3, and tracking performance along *X, Y*, and *Z* directions are shown in Figures 3A–C, respectively. In Figure 3, tracking performance *via* the traditional SMC and SMCNDO are drawn in blue dashed line and red dashed line, respectively, and the predefined trajectory is drawn in black-solid line. The output forces for the three wires in simulation *via* SMC and SMCNDO are shown in Figure 4. Figures 4A–C are the output forces *via* SMC for wire 1, wire 2, and wire 3, respectively. Figures 4D–F are the output forces *via* SMCNDO for wire 1, wire 2, and wire 3, respectively. From Figure 4, we can find that chattering of the output forces *via* SMC is relatively severe, whereas chattering is reduced effectively by implementation of SMCNDO. These simulation results indicate that when control the wire-driven robot follow a predefined trajectory in the presence of unknown disturbances, SMCNDO has better tracking performance and lower chattering compared with the traditional SMC.

**Figure 5:**
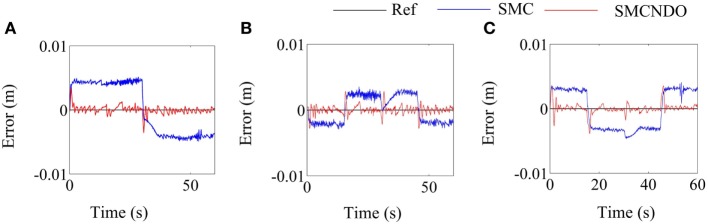
Comparison of tracking errors in the three coordinate directions by implementation of sliding mode control (SMC) and sliding mode control with nonlinear disturbance observer (SMCNDO) when a healthy subject follows a square-shaped trajectory. **(A)** Tracking errors in the *X* direction by implementation of SMC and SMCNDO, **(B)** tracking errors in the *Y* direction by implementation of SMC and SMCNDO, and **(C)** tracking errors in the *Z* direction by implementation of SMC and SMCNDO.

**Figure 6:**
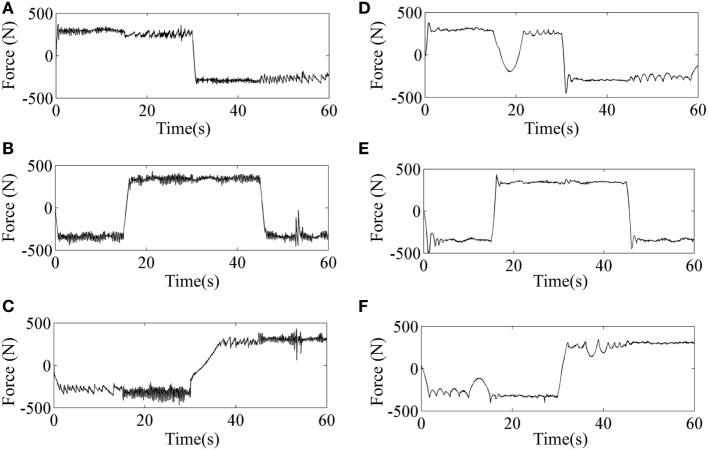
Comparison of output forces for the three wires by implementation of sliding mode control (SMC) and sliding mode control with nonlinear disturbance observer (SMCNDO) when a healthy subject follows a square-shaped trajectory. **(A)** The output force for wire 1 by implementation of SMC, **(B)** the output force for wire 2 by implementation of SMC, **(C)** the output force for wire 3 by implementation of SMC, **(D)** the output force for wire 1 by implementation of SMCNDO, **(E)** the output force for wire 2 by implementation of SMCNDO, and **(F)** the output force for wire 3 by implementation of SMCNDO.

**Figure 7:**
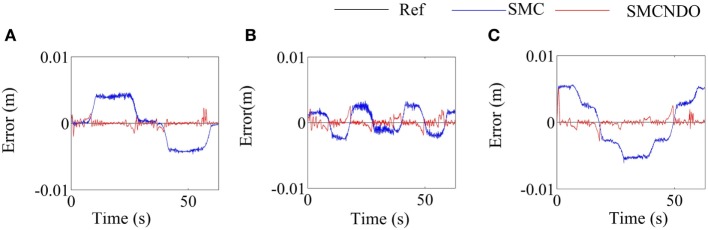
Comparison of tracking errors in the three coordinate directions by implementation of sliding mode control (SMC) and sliding mode control with nonlinear disturbance observer (SMCNDO) when a healthy subject follows a circle-shaped trajectory. **(A)** Tracking errors in the *X* direction by implementation of SMC and SMCNDO, **(B)** tracking errors in the *Y* direction by implementation of SMC and SMCNDO, and **(C)** tracking errors in the *Z* direction by implementation of SMC and SMCNDO.

The tracking errors of the two applied control schemes in the three coordinate directions when a healthy subject follows the predefined square-shaped trajectory are shown in Figure 5, where the tracking errors by implementation of the traditional SMC and SMCNDO are drawn in blue-solid line and red-solid line, respectively, the black-solid line is added as a reference whose value is always 0. From Figure 5, it is observed that SMCNDO has obviously smaller tracking errors along all the three directions. In addition, the root mean square errors along the three coordinate directions of tracking the square-shaped trajectory are calculated based on Eq. 26 and are show in Table 2. It can be found in Table 2 that the root mean square errors of SMCNDO are obviously less than the traditional SMC in all the three coordinate directions. The output forces for the three wires when tracking the square-shaped trajectory by implementation of the traditional SMC and SMCNDO are shown in Figure 6. Figures 6A–C are the results of the output force by implementation of SMC, and Figures 6B,C and 7A are the results of the output forces by implementation of SMCNDO. It can be seen from these plots that the output forces for the three wires by implementation of SMCNDO are much smoother than that of the traditional SMC, which means chattering is effectively reduced by implementation of SMCNDO.

**Table 2:** Root mean square errors (cm) in the three coordinate directions by implementation of sliding mode control (SMC) and sliding mode control with nonlinear disturbance observer (SMCNDO) when a healthy subject follows a square-shaped and a circle-shaped trajectory.

Type of trajectory	*X position*		*Y position*		*Z position*	
	SMC	SMCNDO	SMC	SMCNDO	SMC	SMCNDO
Square-shaped	0.301	0.047	0.177	0.061	0.389	0.071
Circle-shaped	0.411	0.075	0.214	0.066	0.313	0.081

**Figure 8:**
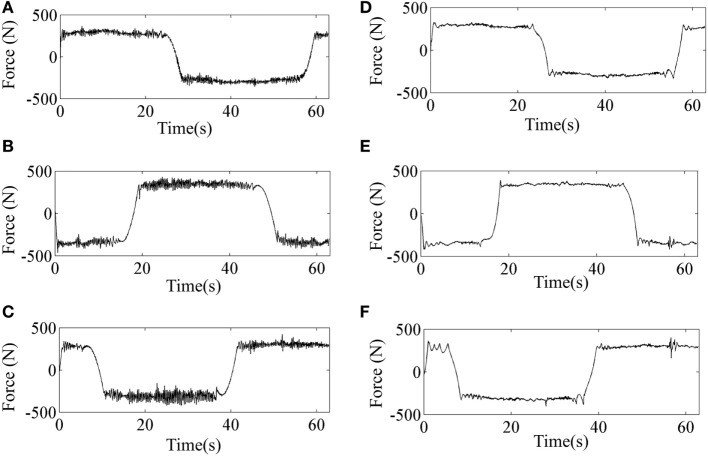
Comparison of the output forces for the three wires by implementation of sliding mode control (SMC) and sliding mode control with nonlinear disturbance observer (SMCNDO) when a healthy subject follows a circle-shaped trajectory. **(A)** The output force for wire 1 by implementation of SMC, **(B)** the output force for wire 2 by implementation of SMC, **(C)** the output force for wire 3 by implementation of SMC, **(D)** the output force for wire 1 by implementation of SMCNDO, **(E)** the output force for wire 2 by implementation of SMCNDO, and **(F)** the output force for wire 3 by implementation of SMCNDO.

The tracking errors along the three coordinate directions when tracking the predefined circle-shaped trajectory by implementation of SMC and SMCNDO are shown in Figure 7. It can be noticed from Figure 7 that SMCNDO has better tacking performance in a three-dimensional space as compared with the traditional SMC. The root mean square errors along the three coordinate directions are also calculated in this experiment according to Eq. 26 and are shown in Table 2, which also indicates that SMCNDO has less tracking errors in the three coordinate directions as compared with the traditional SMC. The output forces for the three wires when tracking the predefined circle-shaped trajectory by implementation of SMC and SMCNDO are shown in Figure 8. Figures 8A–C are the results of the output forces by implementation of SMC, and Figures 8A–C are the results of the output forces by implementation of SMCNDO. From these plots, it can be observed that chattering is effectively reduced by SMCNDO, as the output forces by implementation of SMCNDO is much smoother than that of the traditional SMC.

## Discussion

The designed wire-driven rehabilitation in this paper has three DOF and its architecture makes it possible to assist the subject in performing predefined movements in a three-dimensional space. Coordinate system that applied in the control scheme design procedure is considered in this paper, which can be classified into the wire length coordinate system and the task space coordinate system. In the wire length coordinate system, the length of each wire is measured by scaling the associated motor encoder count; however, due to the unavailable flexibility of wires, using the wire length coordinate system is not reliable when the implementation may require high accuracy. In this paper, this problem can be effectively addressed by using the task space coordinates (25), where the spatial position of the end-effector is measured directly using the motion capture system (OptiTrack, NaturalPoint, USA).

From the results of both simulation and experiments, the traditional SMC has degraded tracking performance and can cause high chattering in all the three coordinate directions, while tracking performance can be obviously improved and chattering can be effectively reduced *via* SMCNDO. The results of experiments are consistent with those of simulation. In literature, many studies have found that the traditional SMC is not able to achieve satisfactory tracking performance and can cause high chattering (29–31). Mohammed et al. have designed SMCNDO for a pneumatic muscle system (28), Deshpande et al. have designed SMCNDO for an active suspension systems (32), the results of these studies show that the tracking performance is obviously improved by SMCNDO as compared with the traditional SMC, which is consistent with the results of our study. By adding the nonlinear disturbance observer to estimate disturbances and feed them back in the control loop, tracking accuracy can be effectively improved. Moreover, since disturbances can be compensated for and high tracking accuracy can be achieved by SMCNDO, the control gain for SMCNDO may be reduced in this study, this could lead to the result of chattering reduction. When delivering robot-assisted rehabilitation training, many unpredictable disturbance scan affect training such as friction caused by actuators (33), human-applied force due to spasticity (34), and uncertainties due to diverse biomechanical variations (20), whereas SMCNDO could effectively reduce the effect of the above-mentioned unpredictable disturbances.

There are a few limitations of this study that should be addressed in the future. The positions of the wires should be well arranged to ensure enough workspace and the safety for upper-limb rehabilitation in clinical application. In this pilot study, we mainly focus on the investigation of the feasibility of the wire-driven rehabilitation robot using SMCNDO, in the future, we will recruit enough patients after stroke to investigate its clinical effectiveness in rehabilitation training.

## Conclusion

In this paper, a wire-driven rehabilitation robot is designed for upper-limb rehabilitation training, and SMCNDO is designed for this robot. Simulation and experimental results of trajectory tracking show that the wire-driven rehabilitation robot with the designed control scheme has exhibited two superiorities including tracking performance improvement and chattering reduction as compared with the traditional sliding mode control scheme. The wire-driven rehabilitation robot with the designed control scheme may have great potential in robot-aided rehabilitation training.

## Ethics Statement

This study was approved by the ethics committee of the Injury Rehabilitation Hospital of Guangdong Province.

## Author Contributions

JN and RS conceived and designed the study. JN and QY performed the experiments, wrote the paper, contributed to the work equally, and should be regarded as cofirst authors. RS reviewed and edited the manuscript. XW made a contribution to experiments. All authors had read and approved the manuscript.

## Conflict of Interest Statement

The authors declare that the research was conducted in the absence of any commercial or financial relationships that could be construed as a potential conflict of interest.

## Abbreviations

SMC, sliding mode control; SMNDO, sliding mode control with nonlinear disturbance observer.
