# Corrigendum: Sliding Mode Tracking Control of a Wire-Driven Upper-Limb Rehabilitation Robot with Nonlinear Disturbance Observer

**DOI:** 10.3389/fneur.2018.00511

**Published:** 2018-06-28

**Authors:** Jie Niu, Qianqian Yang, Xiaoyun Wang, Rong Song

**Affiliations:** ^1^Guangdong Provincial Engineering and Technology Center of Advanced and Portable Medical Devices, School of Engineering, Sun Yat-sen University, Guangzhou, China; ^2^Injury Rehabilitation Hospital of Guangdong Province, Guangzhou, China

**Keywords:** rehabilitation robot, wire-driven, upper limb, tracking control, sliding mode, nonlinear disturbance observer

In the original article, there was an error. The article partly overlaps with previously published conference proceedings. A correction has been made to add an Acknowledgments section.
